# Microstructures and mechanical performance of polyelectrolyte/nanocrystalline TiO_2_ nanolayered composites

**DOI:** 10.1186/1556-276X-8-44

**Published:** 2013-01-21

**Authors:** Bin Zhang, Hai-Feng Tan, Jia-Wei Yan, Ming-Dong Zhang, Xu-Dong Sun, Guang-Ping Zhang

**Affiliations:** 1 Key Laboratory for Anisotropy and Texture of Materials (Ministry of Education), Northeastern University, 3-11 Wenhua Road, Shenyang, 110819, People's Republic of China; 2 Shenyang National Laboratory for Materials Science, Institute of Metal Research, Chinese Academy of Sciences, 72 Wenhua Road, Shenyang, 110016, People's Republic of China

**Keywords:** Bio-inspired nanolayered composite, Layer-by-layer self-assembly, Chemical bath deposition, Mechanical property

## Abstract

Biological materials with hierarchically laminated structures usually exhibit a good synergy between strength and fracture toughness. Here, we show that a bio-inspired (polyelectrolyte (PE)/TiO_2_)_4_ nanolayered composite with a thickness ratio of TiO_2_ and amorphous PE layers of about 1.1 has been prepared successfully on Si substrates by layer-by-layer self-assembly and chemical bath deposition methods. Microstructures of the nanolayered composite were investigated by scanning electron microscopy, secondary ion mass spectroscopy, and high-resolution transmission microscopy. Mechanical performance of the composite was characterized by instrumented indentation. The composite consisting of 17.9-nm-thick nanocrystalline TiO_2_ and 16.4-nm-thick amorphous PE layers has a strength of about 245 MPa, which is close to that of shells, while the fracture toughness of the composite, *K*_IC_ = 1.62 ± 0.30 MPa · m^1/2^, is evidently higher than that of the bulk TiO_2_. A possible strategy to build the composite at nanoscale for high mechanical performance was addressed.

## Background

Biological materials (such as bones or shells, etc.) with multiscale and hierarchical structures consisting of thick, hard inorganic mineral layers and thin, soft organic layers exhibit an excellent combination of strength and toughness [1,2]. Although a number of metallic multilayered/laminated composites produced by various methods [3,4] had revealed a high strength and a potential improvement of plasticity without losing strength owing to the contribution of the laminated structures and interfaces [5], a few efforts had been made to design and synthesize bio-mimetic laminated materials with submicron-thick inorganic layers [6-11]. Theoretically, Gao et al. [12] demonstrated that when the critical length scale of the mineral inorganic platelets in natural materials drops below approximately 30 nm, the biomaterials became insensitive to flaws, i.e., the strength of a perfect mineral platelet was maintained despite defects. This intrigued us to design and synthesize the artificial counterparts of this composite with nanometer-thick constituent layers less than 30 nm.

In this work, a variation method of combination of traditional chemical bath deposition (CBD) [10,13] and layer-by-layer (LBL) self-assembly [14] methods was conducted to prepare a layered structure stacked alternately by nanocrystalline TiO_2_ and polyelectrolyte (PE) layers with thicknesses less than 30 nm. Microstructures and mechanical properties of the nanolayered composites (NLCs) were investigated.

## Methods

Silicon (001) substrates (3 × 10 mm^2^) were immersed in Piranha solution [15] for 20 min at 60°C after ultrasonic cleaning in acetone. A negatively charged hydrophilic Si-OH layer was formed on the Si surface. Owing to the electrostatic attraction of oppositely charged polyions, three different PEs, poly(ethyleneimine) (PEI), poly(sodium 4-styrenesulfonate) (PSS), and poly(allylamine hydrochloride) (PAH), were selected as polycation, polyanion, and polycation, respectively, and the organic polymer layers were assembled by LBL deposition [14] of the three different PEs. The negatively charged Si substrates (after Piranha treatment) were alternately immersed into the three different PE solutions in the sequence (PEI/PSS)(PAH/PSS)_3_[10,14], and the immersion in the respective polymer solutions was at room temperature for 20 min. A positively charged surface was formed by adsorption of PEI on silicon since PEI can give good covering of oxidized surfaces [14]. The thickness of the PE layers was controlled by the number of dipping cycles into PAH/PSS solutions, while three dipping cycles were carried out in the present work to ensure the thickness of the PE layers to be less than 30 nm. Deposition of inorganic TiO_2_ layers onto the PE surface was accomplished in a 10 mM solution of titanium peroxo complex (TiO_2_^2+^) and 30 mM HCl by the CBD procedure [10]. In order to ensure the thickness of the deposited TiO_2_ layer to be less than 30 nm, the adopted deposition time and temperature were 2 h and 60°C, respectively. The PE/TiO_2_ NLCs with four bilayered periods ((PE/TiO_2_)_4_) were prepared finally by sequentially applying the LBL self-assembly and the CBD techniques.

Secondary ion mass spectroscopy (SIMS; ION-TOF TOF.SIMS 5, Münster, Germany) was utilized to determine the existence of Ti, O, C, and Si ions, as a function of depth below the film surface. A nanoindenter (Hysitron TI 900, Eden Prairie, MN, USA) with a Berkovich tip (tip radius approximately 50 nm) was used to determine the hardness (*H*) and modulus (*E*) of the NLCs at a constant loading rate of 20 μN/s at room temperature. The mean values of *H* and *E* were then obtained at an indentation depth of 10% to 20 % whole thickness of the NLC in order to eliminate substrate effects [16]. Microindentation tests (LECO AMH43, St. Joseph, MI, USA) were conducted to evaluate fracture toughness of the NLCs following the method proposed by Xia et al. [17].

## Results and discussion

### Microstructures

**Figure 1:**
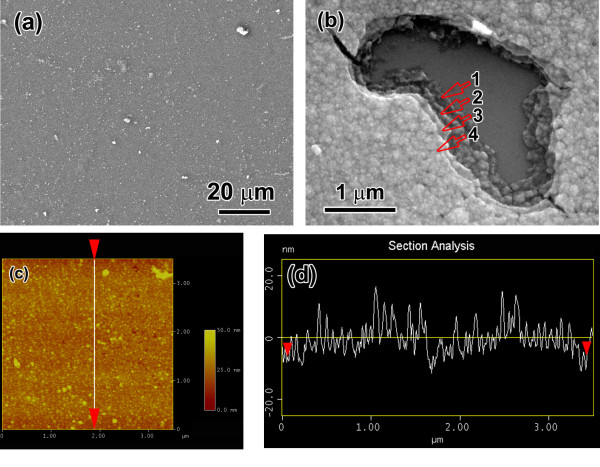
**SEM observations, AFM characterization, and surface roughness of the nanocomposite.** SEM observations on surface of the (PE/TiO_2_)_4_ nanolayered composite: **(a)** surface morphology and **(b)** layer structure. **(c)** AFM characterization of surface of the nanocomposite. **(d)** Surface roughness of the nanocomposite measured by AFM.

A scanning electron microscopy (SEM) observation (Figure 1a) shows that the surface of the (PE/TiO_2_)_4_ NLC is quite smooth. A cracking region caused by a scratching of a needle reveals that the NLC is a typical multilayered structure with four layers, as indicated by arrows in Figure 1b. The surface morphology of the NLC examined by atomic force microscopy (Figure 1c) shows that the top TiO_2_ layer is a densely packed spherical particle with a diameter of approximately 40 nm. The surface roughness of the top TiO_2_ layer is about 4.5 nm (Figure 1d).

**Figure 2:**
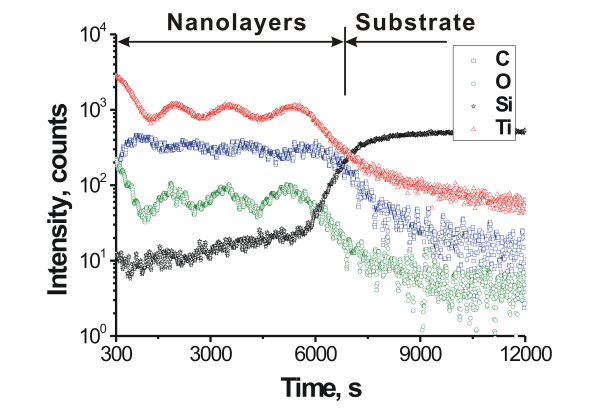
**SIMS characterizations.** Variation of the intensity of ejected secondary ions of the present elements as a function of sputtering time of primary ion beam characterized by secondary ion mass spectroscopy.

SIMS characterizations of the intensity variations of the ejected secondary ions of the present elements as a function of sputtering time of the primary ion beam exhibit that there is a periodical variation of the intensity of O ion and Ti ion with the sputtering time (Figure 2), while the intensity of C ion exhibits an inverse periodical variation with the sputtering time. After the appearance of four peaks of the periodical variation of the elements, the intensity of the Ti and C ions becomes decreased, while that of the Si ion becomes strong and finally reaches a certain intensity level, indicating the appearance of the Si substrate. The profile clearly demonstrates the presence of a multilayered structure of alternating TiO_2_-enriched and C-enriched layers, i.e., the existence of an ordered composite structure of well-defined inorganic and organic layers.

**Figure 3:**
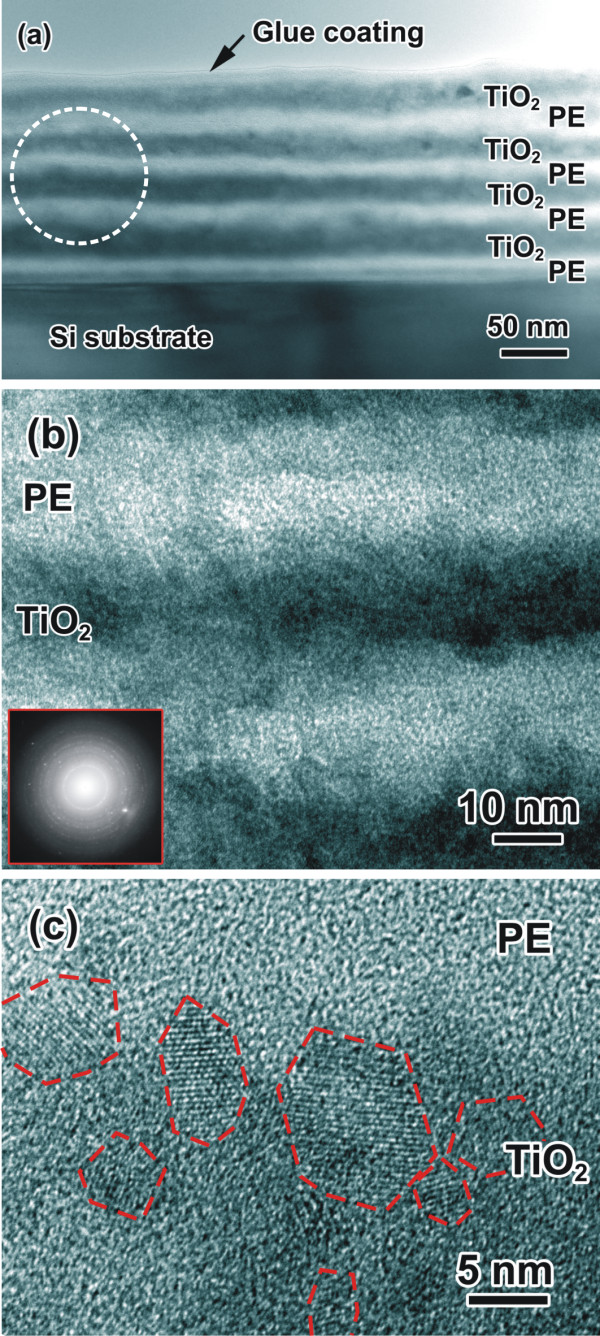
**TEM cross-sectional images of the composite and HRTEM image of the interface.** TEM cross-sectional images of the (PE/TiO_2_)_4_ nanolayered composite at **(a)** low magnification and **(b)** high magnification. **(c)** HRTEM image of inorganic TiO_2_ layer and organic/inorganic interface.

A transmission electron microscopy (TEM) cross-sectional observation at a low magnification (Figure 3a) also clearly reveals the multilayered structure in the (PE/TiO_2_)_4_ NLC, though there is interpenetration between the PE and TiO_2_ layers (see Figure 3b). The organic PE layers appear as bright regions with an average thickness of 16.4 nm, while the inorganic TiO_2_ layers are visible as dark regions with an average thickness of 17.9 nm estimated from TEM cross-sectional images. Thus, the thickness ratio (*R*_t_) of the TiO_2_ layer to the PE layer is about 1.1. A high magnification of the PE/TiO_2_ NLC (Figure 3b) shows that the interface between the PE and TiO_2_ layers is not sharp completely, but somewhat diffuse, indicating a sizeable interpenetration between the TiO_2_ and organic PE components [10]. A selected-area electron diffraction pattern taken from the dotted-circle region in Figure 3a was presented in the inset of Figure 3b, revealing the diffuse diffraction ring corresponding to the amorphous PE layers, while some diffraction spots exhibit the existence of crystallites. A high-resolution transmission electron microscopy (HRTEM) image (Figure 3c) shows that some nanocrystallines (NCs) with different orientations have formed in the TiO_2_ layer and their sizes are in a range of about 5 to 15 nm. The NC TiO_2_ might form during the CBD process rather than the TEM electron-beam irradiation since the TEM accelerating voltage we used was 200 keV rather than 400 keV [10]. The formation of the NC TiO_2_ might be related to the very thin TiO_2_ layers (approximately 17.9 nm) deposited in a short time (2 h) of the CBD process. In addition, the rough and thin PE layers assembled by few numbers of cycles (3 cycles) for the PAH/PSS might also play an important role in the heterogeneous nucleation of the TiO_2_ nanocrystallines.

### Mechanical performance

**Figure 4:**
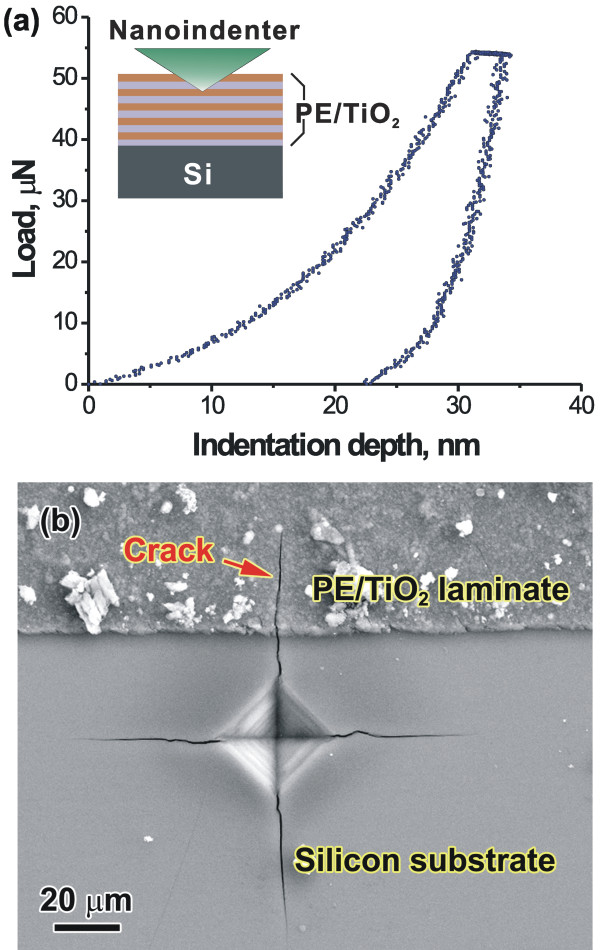
**Load-indentation depth curve of the composite and SEM image of the indentation-induced microcrack. (a)** Load-indentation depth curve of the (PE/TiO_2_)_4_ nanolayered composite measured by nanoindentation. **(b)** SEM image showing that indentation-induced microcrack advanced into the (PE/TiO_2_)_4_ nanolayer-coated region, by which fracture toughness of the nanocomposite can be obtained.

Figure 4a shows a typical load-indentation depth curve of the (PE/TiO_2_)_4_ NLC. In the loading stage, no pop-in behavior was detected, indicating that the NLC can be deformed continuously to the indentation depth of about 30 nm. In the unloading stage, the initially linear unloading reveals an elastic recovery. With a further unloading, the nonlinear variation of the load with the displacement reveals the non-elastic recovery, leading to a residual indentation depth of about 22 nm. Young's modulus of the NLC determined from the contact area and the elastic contact stiffness [16] is 17.56 ± 1.35 GPa, which is much lower than that of the nacre (*E* = 50 GPa) [18]. Such a low Young's modulus may be attributed to the large volume fraction of organic PE layers due to *R*_t_ ≈ 1.1. Based on the rule of mixture, Young's modulus is estimated to be about 16.74 GPa by using $E_{\mathrm{Ti}O_{2}}$ = 27.5 GPa and *E*_PE_ = 5 GPa [11], and this is close to the experimental result of the (PE/TiO_2_)_4_ NLC (17.56 GPa). The mean hardness of the (PE/TiO_2_)_4_ NLC determined by nanoindentation is 0.73 GPa with a standard deviation of 0.09 GPa. Using a general relation between hardness (*H*) and strength (*σ*) found in a lot of materials, σ≅H3, the mean strength of the NLC was calculated as about 245 MPa, which is quite close to the strength of shells reported in the literature (100 to 300 MPa) [10,18]. Although *R*_t_ ≈ 1.1, it is expected that the NC TiO_2_ layers would also contribute to the high strength of the composite.

Following the method to determine the fracture toughness (*K*_IC_) of a thin film bonded to a brittle substrate [17], when the indentation load was large enough applied to the Si substrate uncoated by the (PE/TiO_2_)_4_ NLC, microcracks initiated from four corners of the indent in the Si substrate and advanced into the (PE/TiO_2_)_4_ nanolayer-coated region, as indicated by an arrow in Figure 4b. Based on the measurements of the crack length, *K*_IC_ of the (PE/TiO_2_)_4_ NLC was obtained as *K*_IC_ = 1.62 ± 0.30 MPa · m^1/2^, which is almost a threefold increase in comparison to that of the single TiO_2_ layer of approximately 400 nm thick [11]. One reason for the enhancement of *K*_IC_ of the present NLC was attributed to energy dissipation via crack deflection along the inorganic/organic interface, as a general mechanism operated in artificial and natural multilayered architectures [11]. Furthermore, since the present (PE/TiO_2_)_4_ NLC has an inorganic/ organic layer thickness ratio of about 1.1 and the TiO_2_ thickness is only 17.9 nm, it is believed that even if a crack initiates in the TiO_2_ layer with a thickness of 17.9 nm, the NLC would become more insensitive to flaws, as predicted by Gao et al. [12]. The hierarchical structures in biological materials have shown a good synergy of high strength and good fracture toughness (damage tolerance). Li et al. [19] have revealed that the mineral layer in the nacre consists of nanocrystalline CaCO_4_ platelets, which facilitates grain boundary sliding. This also implies the possible activation of the grain boundary sliding mechanism in our NC TiO_2_ layers during deformation. The present results indicate that building the composite consisted of the amorphous PE and the NC TiO_2_ layers at nanometer scales may provide a possible strategy toward enhancing damage tolerance of the material even if the best optimum ratio of the organic layer to the NC inorganic layer still needs to be found.

## Conclusions

The bio-inspired (PE/TiO_2_)_4_ nanolayered composite with an inorganic/organic layer thickness ratio of about 1.1, which consisted of nanocrystalline TiO_2_ and amorphous PE layers with thicknesses of 17.9 and 16.4 nm, respectively, was prepared on a Si (001) substrate by LBL self-assembly and CBD methods. The (PE/TiO_2_)_4_ nanocomposite has a strength of about 245 MPa, being close to that of the natural shell, while the fracture toughness of the nanocomposite, *K*_IC_ = 1.62 ± 0.30 MPa · m^1/2^, is evidently higher than that of the single TiO_2_ of about 400 nm thick.

## Abbreviations

CBD: Chemical bath deposition; LBL: Layer-by-layer; NC: Nanocrystalline; NLC: Nanolayered composite; PE: Polyelectrolyte.

## Competing interests

The authors declare that they have no competing interests.

## Authors’ contributions

All authors contributed equally to this work. BZ, XDS, and GPZ conceived the project. BZ, HFT, and MDZ performed the experiments. JWY performed the TEM observations. All authors analyzed the data, discussed the results, and wrote the paper. All authors read and approved the final manuscript.
